# Contemporary and novel applications of spinal navigation

**DOI:** 10.3171/2026.4.FOCVID2564

**Published:** 2026-07-01

**Authors:** Paul Park, Dean Chou, Yoon Ha, Corinna C. Zygourakis, Juan S. Uribe

**Affiliations:** 1 Semmes Murphey, Department of Neurosurgery, University of Tennessee, Memphis, Tennessee;; 2 Department of Neurological Surgery, Columbia University Irving Medical Center, New York, New York;; 3 Department of Neurosurgery, Severance Hospital, Yonsei University College of Medicine, Seoul, Korea;; 4 Department of Neurosurgery, Stanford University School of Medicine, Stanford, California; and; 5 Department of Neurosurgery, Barrow Neurological Institute, St. Joseph’s Hospital and Medical Center, Phoenix, Arizona

## INTRODUCTION

Navigation in spinal surgery is well established, and there exists substantial literature to support improved accuracy of pedicle screw placement.^1^ However, the impact of navigation beyond screw placement has not been as well promoted despite growing clinical usage, especially in minimally invasive procedures in which direct visualization is severely limited. Navigation allows accurate target localization and trajectory planning to guide placement of endoscopes and tubular retractors with minimal radiation exposure. It can also facilitate precise removal of bone for decompression and, for deformity cases, assist in osteotomy creation. These expanded applications of navigation technology offer the potential for more consistent and improved outcomes.

*This Neurosurgical Focus: Video* issue highlights a variety of applications using spinal navigation to enhance surgical efficiency and outcomes as well as minimizing complications. Many of the videos in this issue highlight the impact of navigation in minimally invasive lateral surgery. Another video shows the positive impact of navigation on cervical laminoplasty. Other videos illustrate the benefits of robotic navigation in the treatment of degenerative and oncological disease. Potential advantages of navigation and augmented reality for endoscopic surgery are also included.

## Disclosures

P. Park reported royalties from Globus; personal fees from Globus, Medtronic, Kuros, and Cerapedics; stock in ATEC and OnPoint Surgical; and grants from ISSG and Cerapedics outside the submitted work. D. Chou reported personal fees from Medtronic and Globus outside the submitted work. J. Uribe reported personal fees from ATEC outside the submitted work.
